# Primary mesenteric abscess caused by Klebsiella pneumoniae: A case report

**DOI:** 10.1097/MD.0000000000035774

**Published:** 2023-10-27

**Authors:** Peng Wang, Fengfeng Zhu, Mingming Wang, Bingxu Niu, Bin Ma, Jundong Du

**Affiliations:** a Department of General Surgery, Jincheng General Hospital, Shanxi Medical University, Jincheng, China; b Department of General Surgery, Jincheng People’s Hospital, Jincheng, China; c Graduate School, Changzhi Medical College, Changzhi, China; d Department of Oncologic Surgery, Lanzhou University Second Hospital, Lan zhou, China.

**Keywords:** mesenteric abscess, Klebsiella pneumoniae, intraperitoneal infection, complications

## Abstract

**Rationale:**

Mesenteric abscess, a rare abdominal infection, is regularly mostly secondary to inflammatory bowel disease, diverticula of the small intestine, or tuberculosis. Primary mesenteric abscesses are extremely rare. If not diagnosed and treated in a timely manner, it may lead to serious consequences; computerized tomography is highly beneficial for the diagnosis of this disease; timely surgical intervention, judicious use of antibiotics, and adequate nutritional support are crucial in the management of this disease.

**Patient concerns:**

A 59-year-old male patient from China was admitted to hospital for intermittent abdominal pain accompanied by poor appetite for 10 days. One week before admission, the patient had been infected with corona virus disease 2019. Past history includes type 2 diabetes and post-operative gastric cancer.

**Diagnosis:**

The emergency abdominal computerized tomography examination results of the patient suggested that the mesentery was cloudy with a large amount of effusion and visible bubble. Mesentery abscess was considered, but duodenal perforation could not be excluded.

**Interventions:**

We adopted exploratory laparotomy to further clarify the diagnosis. Intraoperatically, after fully exposing the duodenum, we found extensive abscess formation in the mesentery, but no duodenal perforation. After operation, the patient developed duodenal leakage and was treated with gastric tube and jejunal nutrition tube.

**Outcomes:**

Postoperatively, due to poor general condition, the patient was transferred to intensive care unit; after anti-infective treatment, the condition improved on the 5th postoperative day, and duodenal leakage appeared on the 9th postoperative day, and conservative treatment was ineffective, and the patient eventually died.

**Lessons:**

Primary mesenteric abscess is a local tissue infectious disease. Whereas we should consider the physical basic condition of the patient during therapeutic process. We believe adequate postoperative drainage, rational use of antibiotics based on bacterial culture, early ambulation after surgery, and adequate nutritional support might be key points for successful therapy.

## 1. Introduction

Previous studies have shown that Klebsiella pneumoniae (KP) may belong to Gram-negative bacteria, which can be a common opportunistic pathogen in natural environment. The detection rate of carbapenem-resistant KP has increased year by year.^[1,2]^ KP can trigger urinary tract infection, hospital acquired pneumonia, liver abscess etc, leading to sepsis, high mortality. Whereas, mesenteric abscess caused by KP is rarely reported.

Herein, we report the case of a male patient with a primary mesenteric abscess infected by KP. Our findings suggest that before managing patients undergoing surgery of mesenteric abscess, it is important to carefully assess patients systemic symptoms, possible complications.

## 2. Case report

**Figure 1.**
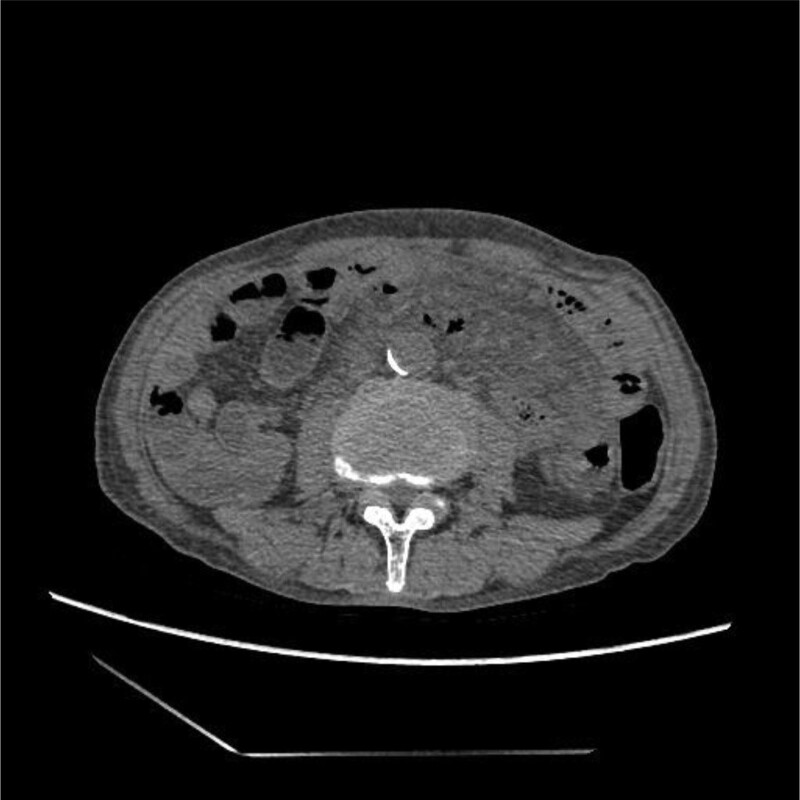
Computerized tomography (CT) showed extensive bubble shadow in mesentery, considering mesenteric abscess, duodenal perforation cannot be excluded.

In the study, a 59-year-old male patient with type 2 diabetes from China underwent radical gastrectomy for proximal gastric cancer 6 years ago. After 6 cycles of chemotherapy according to XELOX regimen, no signs of tumor recurrence and metastasis were found during regular review. In this case, each cycle of the XELOX regimen consisted of oxaliplatin (L-OHP) for 130 mg/m^2^/day for 1 day, combined with capecitabine 1000 mg/m^2^/ day for 14 days. The patient was admitted to hospital for intermittent abdominal pain accompanied by poor appetite for 10 days. One week before admission, the patient had been infected with corona virus disease 2019 (COVID-19), accompanied by intermittent low fever, cough, loss of appetite and other symptoms. However, during this hospitalization, the patient had no accompanying respiratory symptoms such as cough and sputum. After admission, the patient underwent a physical examination. The results showed that the body temperature during admission was 36.5°C and the body mass index was 15.4 kg/m^2^. The patient is in good spirits. On physical examination of both lungs, we found no abnormalities. On physical examination of the abdomen, we found positive for deep tenderness in the upper abdomen, suspicious positive for rebound pain, and active bowel sounds. Therefore, we performed blood-related tests on the patient, and the results pointed 82.60% neutrophils, 1.01 109/L absolute lymphocyte value, 148.35 mg/L C-reactive protein, 3.83 ng procalcitonin/mL, and normal blood glucose. All of the above results indicated elevated inflammatory markers in patients. Furthermore, we performed abdominal computerized tomography (CT) examination of the patient. The results suggested that the mesentery was cloudy with a large amount of effusion and visible bubble. Mesentery abscess was considered, but duodenal perforation could not be excluded (Fig. 1).

**Figure 2.**
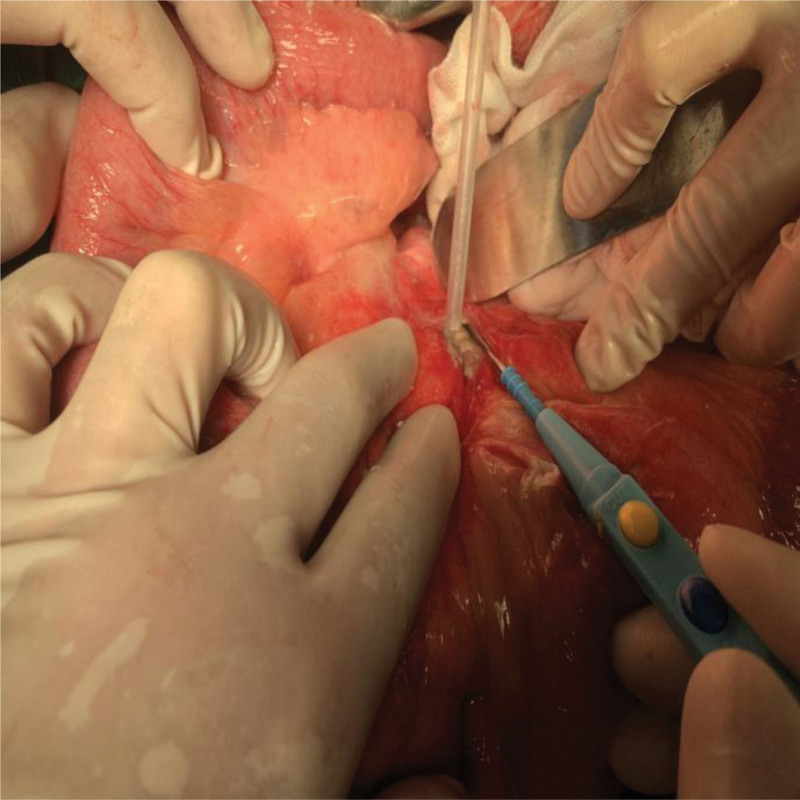
Extensive abscess was found in the mesentery during the operation, with massive yellow turbid abscess visible.

Based on the patient current clinical presentation and auxiliary examination, we considered a mesenteric abscess with a suspicious positive duodenal perforation. Therefore, exploratory laparotomy was adopted. Intraoperatically, after fully exposing the duodenum, we found extensive abscess formation in the mesentery, but no duodenal perforation (Fig. 2). Based on the patient laboratory findings and intraoperative findings, mesenteric abscess incision and drainage were performed. During the operation, it can be found that the pus was pale yellow with a sour odor. Drainage tubes were placed in the mesenteric abscess, pelvic cavity, and splenic fossa. In order to confirm the etiological diagnosis, bacterial culture and abscess wall biopsy was performed on the pus drained during the operation, and the result indicated KP infection. After surgery, the patient was admitted to intensive care unit for further monitoring. In the intensive care unit, 4 g cefoperazone sodium and sulbactam sodium was initially given for anti-infective therapy every 12 hours, and 1 g meropenem was given for anti-infective therapy every 8 hours on the second day after surgery. Fortunately, another laboratory examination on the 5th postoperative day revealed 79% of neutrophils, 60.35 mg/L C-reactive protein, and 1.34 ng/mL procalcitonin. The abdominal drainage can be observed to be yellowish. These above results indicated that the patient infection was under control. Therefore, the intubation was removed.

On the 9th day after surgery, the patient was in critical condition with a large amount of bloody fluid drained from the abdominal cavity. Laboratory examination revealed a dynamic upward trend of inflammation indicators. Bedside digital radiography system showed a whited-out lung on the left side, but no pleural effusion was observed. In addition, the oxygen saturation and blood pressure of the patient indicated a downward trend, so the endotracheal intubation was performed again to set up the respiratory passage and assist respiration. In the following days, the patient drainage fluid gradually became dark red and sticky.

**Figure 3.**
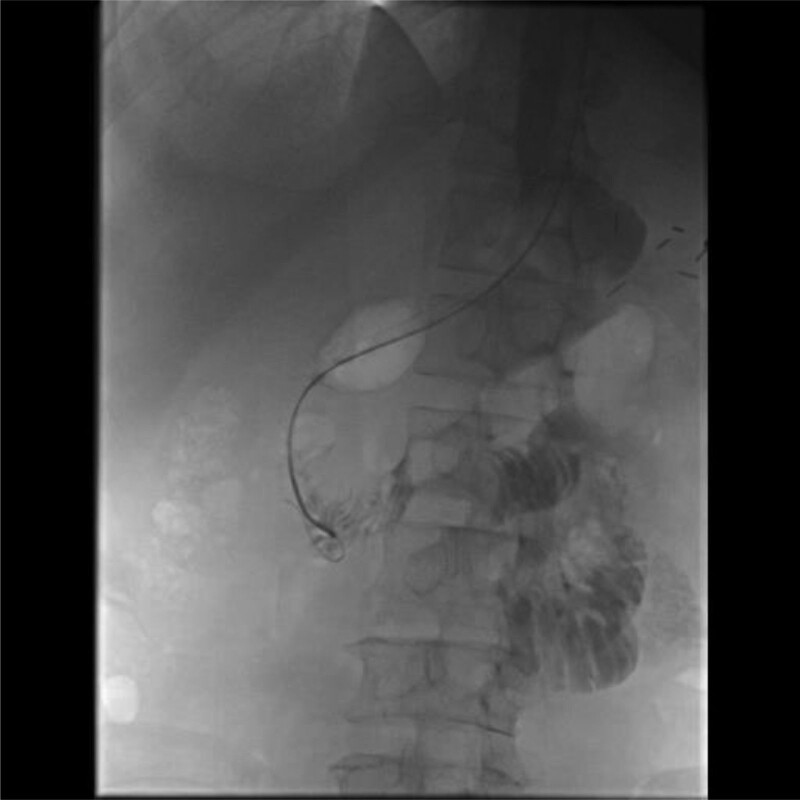
Upper gastrointestinal radiography showed duodenal end leakage.

**Figure 4.**
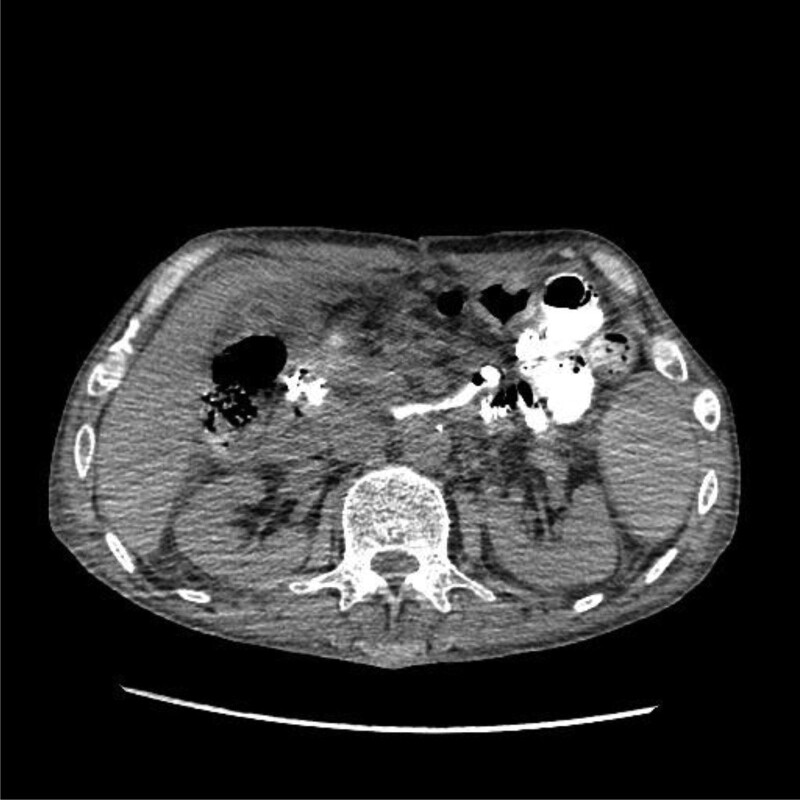
Abdominal computerized tomography (CT) examination was performed 15 minutes after oral contrast agent. Contrast agent can be seen in and around the peritoneal drainage tube, and duodenal leakage was considered.

On the 16th day after operation, the patient peritoneal drainage fluid turned dark green. Therefore, we conducted bacterial culture and drug sensitivity test on the drainage fluid and sputum again, and the results indicated that it was caused by carbapenem-resistant KP infection. The drug sensitivity results showed that the bacteria was sensitive to tegacyclin, so tegacyclin was added to the original treatment scheme (100 mg for the first dose, 50 mg every 12 hours after the treatment). Then we successively performed digestive tract radiography and abdominal CT examination, which indicated duodenal leakage (Figs. 3 and 4). In view of the patient serious condition, we took conservative treatment, and put a jejunal nutrition tube under intervention, with the distal end of the nutrition tube crossing the leakage more than 40 cm. During the treatment of enteral nutrition support, the peritoneal drainage was still dark green, but the drainage volume did not increase.

Unfortunately, on the 24th day after surgery, the patient died due to worsening pulmonary infection and septic shock.

## 3. Discussion

This study reported a case of mesenteric abscess triggered by KP. As far as we know, this might be the first report on mesenteric abscess caused by KP. In this case, the patient mesenteric abscess was not secondary to gastrointestinal perforation, which was extremely rare.

The American Society of Surgical Infections for the Management of Intra-abdominal Infections (2017 Revision) has pointed out that,^[3]^ complex intra-abdominal infection might refer to the infection that breaks through the primary affected organ and enters the abdominal cavity, causing peritonitis or abdominal abscess, which is mostly secondary and often requires surgical intervention. Despite the clinical heterogeneity, the core features of intra-abdominal infection can be relatively common, including anatomical destruction and polymicrobial infection.^[4]^ In 2018, according to the data of the Chinese Antimicrobial Resistance Surveillance of Nosocomial Infections based on the Chinese Hospital Infection Surveillance System,^[5]^ Gram-negative bacteria accounted for 70.8%, gram-positive bacteria accounted for 29.2%, and the most common bacteria were *Escherichia coli* (33.4%), KP (10.8%) and *Enterococcus faecalis* (10.7%). In recent years, the detection rate of KP in the community has also been rising, which has brought great challenges to public health.

The pathogenesis of mesenteric abscess formation is still unclear. The patient was infected with COVID-19 before admission, and there may be a certain relationship between COVID-19 infection and mesenteric abscess. It has been reported that^[6,7]^ COVID-19 infection can lead gastrointestinal symptoms including intestinal perforation. Nevertheless, our patient postoperative intestinal perforation was not consistent with the above situation. In addition, angiotensin converting enzyme 2 protein was the host cell receptor of SARS-CoV-2 virus, which was highly expressed in intestinal epithelium, and was the possible mechanism of SARS-CoV-2 infection in gastrointestinal tract.^[8]^ Furthermore, according to the patient past medical history and the medical history of this admission statement, the patient immune system was further damaged under multiple adverse factors such as previous tumor treatment, insufficient nutrition intake in daily life, and COVID-19 infection.^[9]^ This might provide conditions for the invasion of opportunistic pathogenic bacteria KP. Finally, although the admission blood sugar of the case was normal this time, we can not dismiss the patient history of diabetes and long-term poor glycemic control. The long-term hyperglycemia of patient outside the hospital may inhibit the adhesion, chemotaxis and phagocytosis of leukocytes, and reduce the cellular and humoral immune responses. Previous studies have shown that hyperglycemic environment can facilitate the growth of bacteria in tissues, affect the metabolism of liver, pancreas and gastrointestinal tract, and induce the occurrence of abscess. Long-term poor blood glucose control resulted in abnormal intima of blood vessels, which increased the risk of KP infection.^[10]^

In terms of treatment, we chose the surgical method of exploratory laparotomy, which was consistent with the view of the guidelines for the management of intra-abdominal infection of the American Society of Surgical Infections.^[3]^ Moreover, the progressive mesenteric abscess may compress or even corrode mesenteric vessels, resulting in intestinal ischemia and necrosis or massive bleeding, and even life-threatening. Then, we did not choose conservative treatment. Why don’t we choose ultrasound-guided puncture drainage? On the one hand, the mesentery was rich in blood supply. On the other hand, the patient with thin and weak physical body had a history of gastric cancer surgery, which may trigger serious changes in the position of organs in the abdominal cavity. The ultrasound-guided puncture drainage may lead to side damage. We carried out bacterial culture on pus, which was sensitive to multiple antibiotics at first, and developed into multi-resistant bacteria including carbapenem-resistant bacteria during treatment. One of the reasons was that some KP changed its corynebacterium morphology with the change of environment, enabling the bacteria to escape the recognition and attack of complement in the host.^[11]^ In addition, the patient suffered from intestinal leakage after operation, which we considered to be related to the patient lack of postoperative activities, inadequate drainage of pus, and then the accumulated pus corrodes the blood vessels or intestines, eventually leading to perforation of the intestinal wall. Furthermore, bacterial culture and drug sensitivity tests showed that KP became resistant and antibiotics could not be adjusted in time. In addition, we believed that abscess wall biopsy should also be performed, which can help to exclude tuberculosis^[12]^ and some special types of mesenteric malignant tumors.

Unfortunately, the patient in this study finally died. Whereas, this case still had experience worth summarizing. We analyzed that the failure of treatment may be caused by inadequate debridement or drainage, low albumin level, poor nutritional status, presence of complications and organ dysfunction, severe illness (acute physiology and chronic health evaluation II score ≥ 15), malignant tumor, advanced age, delayed initial intervention (>24 hours), involvement of peritoneum or diffuse peritonitis.^[13,14]^

In summary, there might be no typical clinical manifestation and therapy strategy for mesenteric abscess. There can be some worth remembering experiences for mesenteric abscess treatment, including preoperative CT plain scan and enhanced examination, adequate postoperative drainage, rational use of antibiotics based on bacterial culture, early ambulation after surgery, and adequate nutritional support.

## 4. Conclusion

In the course of treatment, the patient systemic condition was poor, accompanied by hypoproteinemia and multiple organ dysfunction, which may accelerate therapy failure. In the future, in addition to treating the primary disease, patients systemic symptoms, possible complications and healthy education also should be the focus of physician attention. At the same time, we should also recognize our limitations, and we will join forces with multiple centers to share cases and summarize experiences, in the hope of improving the clinical efficacy of patients with this condition.

## Author contributions

**Writing – original draft:** Peng Wang.

**Writing – review & editing:** Jundong Du, Fengfeng Zhu, Mingming Wang, Bingxu Niu, Bin Ma.
